# Pancreas‐preserving resection of lower biliary tract adenocarcinoma: A coring‐out technique

**DOI:** 10.1002/ags3.12021

**Published:** 2017-07-20

**Authors:** Yasunori Nishida, Motokazu Sugimoto, Motohiro Kojima, Naoto Gotohda, Masaru Konishi, Shinichiro Takahashi

**Affiliations:** ^1^ Department of Hepatobiliary and Pancreatic Surgery National Cancer Center Hospital East Kashiwa Japan; ^2^ Division of Pathology Research Center for Innovative Oncology National Cancer Center Hospital East Kashiwa Japan

**Keywords:** cholangiocarcinoma, pancreas‐preserving resection, transduodenal approach

## Abstract

Surgical resection for distal cholangiocarcinoma is usually carried out using pancreaticoduodenectomy (PD). However, because PD is a complex procedure with a high rate of postoperative complications, the surgical indications should be carefully considered, especially for patients with a decreased performance status, significant comorbidities, and/or anatomical anomalies. If curatively carried out, a less invasive, local resection may be an alternative procedure for such patients. In the current study, we present pancreas‐preserving resection of the lower biliary tract in a patient with early‐stage distal cholangiocarcinoma. This procedure was selected to avoid PD with arterial reconstruction because of arterial anomalies. After an abdominal exploration, a cholecystectomy was carried out and the common hepatic duct was transected. The bile duct was dissected from the pancreatic parenchyma without pancreatic resection, downward to the biliopancreatic ductal confluence. Next, a duodenotomy was done opposite Vater's ampulla. The duodenal mucosa around Vater's ampulla was incised and dissected, and the main pancreatic duct (MPD) was divided. The bile duct was completely separated from the pancreatic parenchyma, and the lower biliary tract was totally “cored‐out”. After resection, the MPD was re‐implanted into the duodenal wall, and the duodenotomy was closed. Finally, a Roux‐en‐Y hepaticojejunostomy was created. Postoperative course was uneventful. No tumor recurrence has been observed for 21 months after the operation. Thus, pancreas‐preserving resection of the lower biliary tract appeared to be appropriate for our patient. This organ‐preserving approach can be a useful, alternative procedure in selected patients.

## 1 INTRODUCTION

Although cholangiocarcinoma has been a relatively uncommon tumor, the number of newly diagnosed patients is increasing worldwide.^1^ Even with recent advances in chemotherapy, only surgical resection can provide a chance of cure for cholangiocarcinoma.^2^, ^3^, ^4^ For patients with a cholangiocarcinoma located on the distal side of the biliary tract, pancreaticoduodenectomy (PD) is usually indicated.^5^ However, given the complexity of the procedure and the high rate of postoperative morbidity,^6^, ^7^, ^8^ indications for PD should be carefully considered, especially for patients with a decreased performance status, significant comorbidities, and/or anatomical anomalies. If curatively carried out, a less invasive, local resection may be an alternative procedure for such patients. In this report, we present a surgical procedure of pancreas‐preserving resection for a T1 distal cholangiocarcinoma.

## 2 MATERIALS AND METHODS

### 2.1 Case presentation

**Figure 1:**
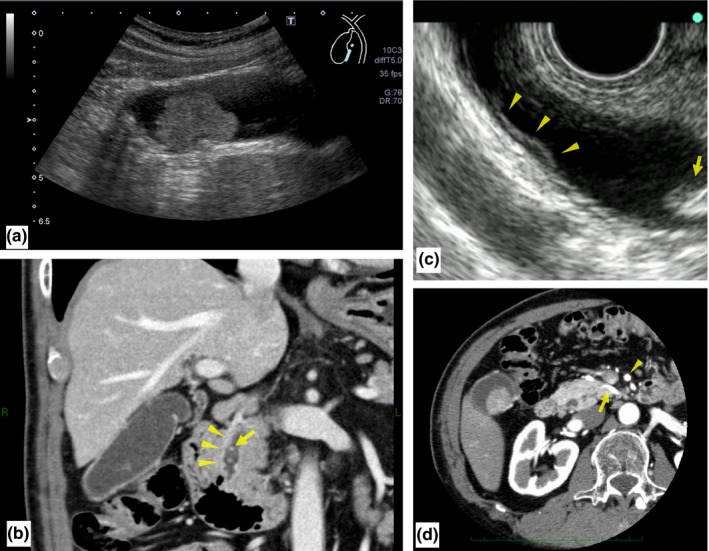
Preoperative images of the present patient. (A) Abdominal ultrasonography shows a papillary tumor in the gallbladder. (B) Abdominal computed tomography (CT) shows small stones (arrow) and the wall thickness of the intrapancreatic bile duct (arrowhead). (C) Endoscopic ultrasonography shows small stones (arrow) and mild irregularity of the mucosa in the lower bile duct (arrowheads). Combined with detection of adenocarcinoma by biopsy, this lesion was diagnosed as a T1 distal cholangiocarcinoma. (D) Abdominal CT shows the replaced common hepatic artery (arrow) originating from the superior mesenteric artery (arrowhead) and coursing along the ventral side of the pancreatic head parenchyma.

**Figure 2:**
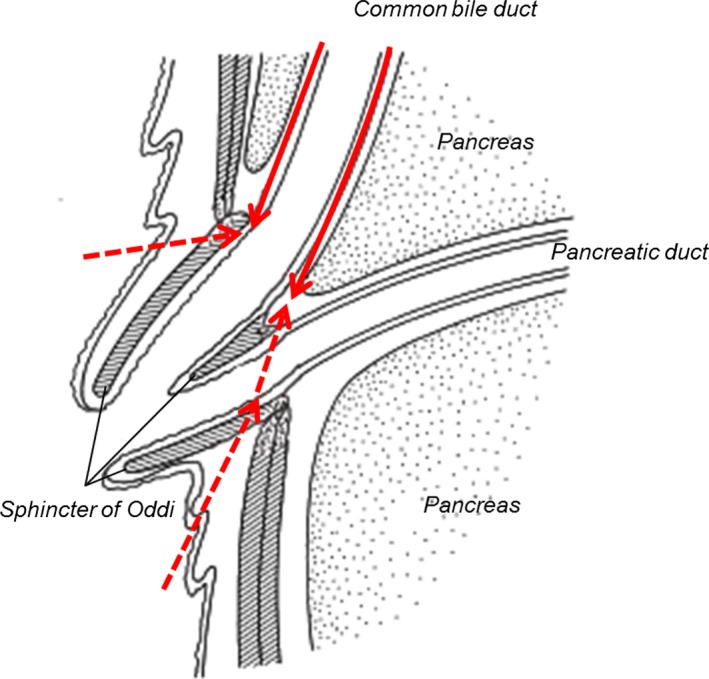
Schematic diagram of pancreas‐preserving resection of the lower biliary tract: use of a coring‐out technique. Red solid line indicates the dissection line downward to the confluence of the bile duct and pancreatic duct (the process is shown in Figure 3A). Red dotted line indicates the dissection line around the biliopancreatic confluence using a transduodenal approach (the process is shown in Figure 3B, C).

A 77‐year‐old, otherwise healthy, man initially presented to a local hospital with upper abdominal pain, where a mass in the gallbladder was detected using computed tomography (CT). He was referred to our hospital for treatment of the gallbladder tumor. Ultrasonography revealed a papillary tumor on the peritoneal side of the gallbladder (Figure 1A). Abdominal multi‐detector CT showed mild wall thickening of the lower common bile duct (CBD) (Figure 1B), in addition to the gallbladder tumor. Endoscopic ultrasonography (EUS) showed multiple small stones and mild irregularity of the mucosa in the lower CBD (Figure 1C). The CBD stones were removed endoscopically, and the duct wall mucosa was biopsied. Pathological specimen from the lower CBD revealed the presence of adenocarcinoma. Preoperative diagnoses confirmed or estimated from imaging exams were as follows: a cholangiocarcinoma with horizontal extension limited within the lower CBD and with vertical extension confined at most to the muscular layer of the CBD (T1), and a gallbladder cancer localized to the peritoneal side of the gallbladder with vertical extension confined at most to the subserosal layer of the gallbladder (T2). No evidence of lymph node metastasis was shown. No continuity was shown between the two lesions by EUS (Figure 1A). Moreover, the arterial phase of the abdominal CT revealed a replaced common hepatic artery (CHA) originating from the superior mesenteric artery and coursing along the ventral side of the pancreatic head parenchyma (Figure 1D). During operative planning, PD with conservation of the replaced CHA was expected to be technically complicated, possibly increasing the risk of postoperative complications, compared with an ordinary PD. Based on detailed preoperative evaluation, PD with arterial resection and reconstruction was also expected to be complex and excessive for resection of the early‐stage tumors. Therefore, we decided on a less invasive procedure for this patient, and carried out pancreas‐preserving resection of the lower biliary tract adenocarcinoma (Figure 2) and cholecystectomy with standardized D2 lymph node dissection.

### 2.2 Surgical techniques

**Figure 3:**
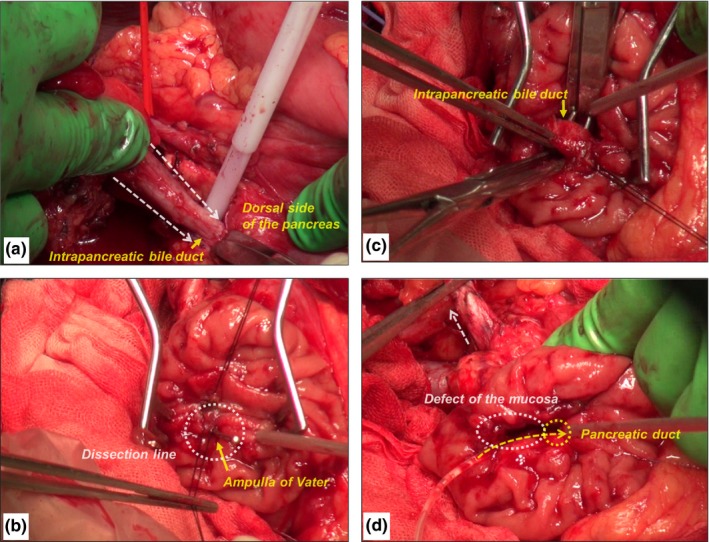
Surgical view: pancreas‐preserving biliary tract resection using a “coring‐out” technique. (A) The bile duct was dissected from the pancreatic parenchyma without pancreatic resection or division, downward to the confluence of the bile duct and main pancreatic duct (MPD). (B) Stay sutures were placed on each side of Vater's ampulla (to be resected) and on the duodenal mucosa (to be preserved). The duodenal mucosa was incised and dissected using electrocautery between each side of the stay sutures. (C) The dissection was continued upward until the bile duct was completely separated from the pancreatic parenchyma. (D) After the extrahepatic bile duct through to Vater's ampulla was totally “cored‐out”, the divided orifice of the MPD was exposed.

Under general endotracheal anesthesia, an upper abdominal mid‐line incision was made (Videos S1 and S2). A generous Kocher maneuver was done to completely mobilize the second part of the duodenum and the head of the pancreas. After cholecystectomy, the common hepatic duct was transected just caudal to the confluence of the right and left hepatic ducts, along with dissection of the lymph nodes around the common hepatic artery and hepatoduodenal ligament. After retropancreatic lymph node dissection, the bile duct was dissected from the pancreatic parenchyma without pancreatic resection or division, downward to the confluence of the bile duct and pancreatic duct (Figure 3A). Next, a 4 cm incision was made on the antimesenteric side of the duodenal wall (opposite Vater's ampulla). We carried out a longitudinal, oblique duodenotomy to avoid narrowing the lumen of the duodenum after closure. Stay sutures were placed on each side of Vater's ampulla (to be resected) and on the duodenal mucosa (to be preserved). The duodenal mucosa was incised and dissected using electrocautery between each side of the stay sutures (Figure 3B). During the dissection, the confluence of the main pancreatic duct (MPD) with the bile duct was identified, and the MPD was divided. The dissection was continued upward until the bile duct was completely separated from the pancreatic parenchyma (Figure 3C). After the extrahepatic bile duct through to Vater's ampulla was totally “cored‐out”, the divided orifice of the MPD was exposed (Figure 3D).

**Figure 4:**
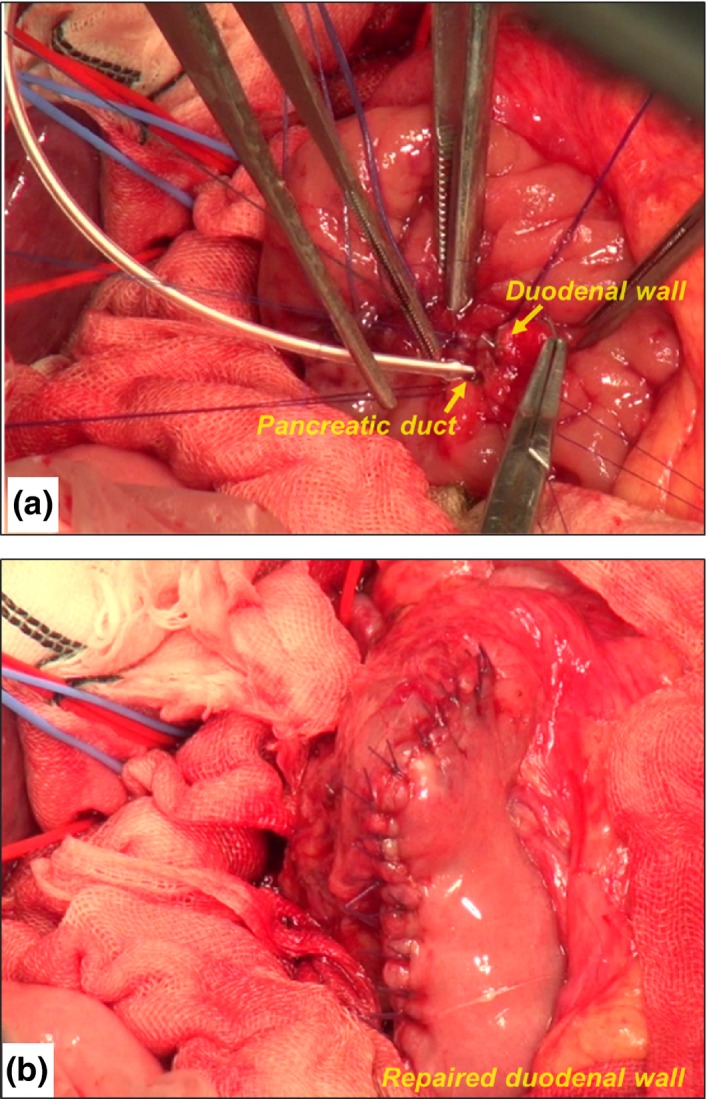
Surgical view: reconstruction. (A) The main pancreatic duct was re‐implanted into the duodenal wall using interrupted 5‐0 absorbable monofilament sutures. (B) The duodenotomy was repaired using the Gambee technique with 4‐0 absorbable sutures.

After resection, the defect in the duodenal wall was identified. This defect was much larger than the size of the MPD, as the biliary tract penetrating the duodenum had been resected en bloc. The defect was closed using interrupted 4‐0 absorbable sutures, leaving a small hole suitable for anastomosis with the MPD. Then, the MPD was re‐implanted into the duodenal wall using interrupted 5‐0 absorbable monofilament sutures (Figure 4A). The sutures incorporated the full thickness of the MPD and the duodenal wall, including the sphincter of Oddi. A short internal drainage tube (lost stent) was inserted through the pancreatoduodenal anastomosis. The duodenotomy was repaired using the Gambee technique with 4‐0 absorbable sutures (Figure 4B). Finally, a Roux‐en‐Y hepaticojejunostomy was created.

## 3 RESULTS

**Figure 5:**
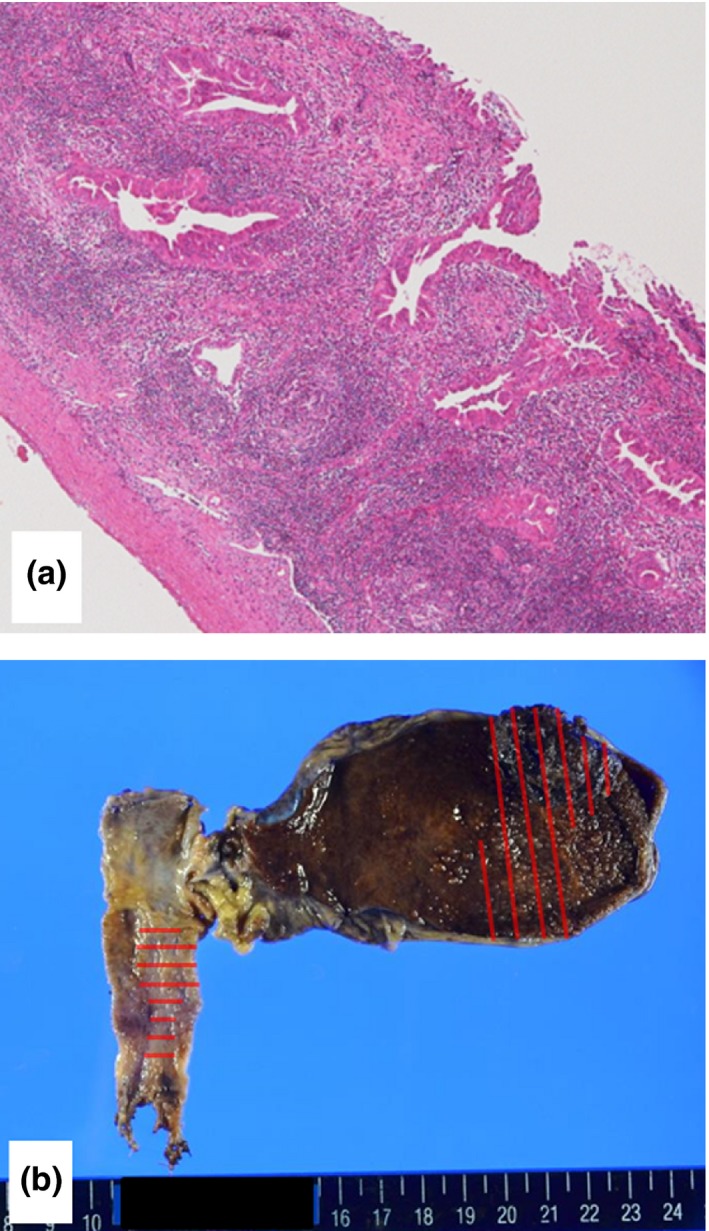
Histological findings. (A) Moderately differentiated adenocarcinoma cells were shown to be confined to the bile duct. (B) The tumor extended horizontally close to Vater's ampulla. All cut end margins and dissected margins were negative for tumor cells.

Operation time was 335 minutes, and estimated blood loss was 468 mL. Postoperative course of the patient was uneventful. An upper gastrointestinal (GI) series on postoperative day 8 demonstrated smooth passage of the contrast material through the entire duodenum to the jejunum. Histological examination of the surgical specimen revealed the two pathologies: a cholangiocarcinoma with stage IA of T1N0M0 (Figure 5A) and a gallbladder cancer with stage II of T2N0M0, according to the American Joint Committee on Cancer Control (AJCC) TNM classification.^9^ The tumor extended horizontally close to Vater's ampulla (Figure 5B); however, all cut end margins and dissected margins were negative for tumor cells. No evidence of tumor recurrence has been seen for 21 months after the operation.

## 4 DISCUSSION

Pancreaticoduodenectomy is a standard surgical procedure for resection of distal cholangiocarcinoma.^5^ However, PD is still associated with a high morbidity rate, despite recent advances in surgical procedures and perioperative management techniques;^6^, ^7^, ^8^ the high degree of surgical invasiveness also leads to a prolonged recovery after surgery. Moreover, considering the function of the pancreas, its resection can lead to endocrine and/or exocrine insufficiency, resulting in diabetes, steatorrhea, and/or malnutrition. Therefore, patients may benefit greatly from a pancreas‐preserving approach in selected cases.

In the present case, we carried out pancreas‐preserving resection of a lower biliary tract adenocarcinoma. A few previous reports on pancreas‐preserving resections of the lower biliary tract have been made. Kondo et al.^10^ reported a case of pancreas‐preserving distal bile duct resection for a patient with a cholangiocarcinoma that had spread in an intraepithelial manner. They divided the pancreatic neck and inserted a thin drainage tube into the main pancreatic duct to achieve complete resection of the biliary system, which required a complicated reconstruction and was likely associated with a high risk of postoperative complications, including postoperative pancreatic fistula. Kolb et al.^11^ reported an organ‐preserving procedure for resection of an intrapancreatic neuroendocrine tumor that involved transection of the dorsal side of the pancreas and closure of the pancreatic parenchyma. The patient received octreotide s.c. for 5 days. In addition, Kim et al.^12^ reported a left hemihepatectomy, caudate lobectomy, and complete extrahepatic bile duct resection using a transduodenal approach in a patient with a hilar cholangiocarcinoma and extrahepatic biliary papillomatosis to avoid an extended hepatectomy combined with PD. Although they carried out the bile duct resection without pancreatic division, they approximated the defect around the excised retropancreatic portion of the CBD. Also, Aiura et al.^13^ reported a slightly different technique for resection of a Vater's ampulla tumor in a high‐risk patient. They injected a saline solution containing diluted epinephrine into the submucosal layer to facilitate dissection of Vater's ampulla, and carried out bile duct resection without pancreatic resection or division or reapproximation of the retropancreatic defect. In the present case, we carried out complete resection of a lower biliary tract adenocarcinoma using a similar approach to Aiura et al., without injecting saline into the periampullary space. As Aiura et al. surmised that dissection to reach around Vater's ampulla from the hepatic side of the bile duct might facilitate the procedure more easily, we first dissected from the hepatic side downward to identify the confluence of the bile duct and the pancreatic duct. Our procedure seemed to be simpler and less invasive than the previously reported pancreas‐preserving approaches.

To determine the indications for this pancreas‐preserving resection, presence of malignancy and tumor extent should be thoroughly assessed using preoperative imaging modalities and intraoperative exploration. This procedure should only be used for patients with benign lesions, premalignant lesions, or locally confined (T1) malignancies of the lower biliary tract, because a T1 distal cholangiocarcinoma rarely has lymph node metastasis.^14^ Moreover, as operative candidates of PD should have a preserved performance status without extensive comorbidities, this procedure is also useful for patients with high operative risk who might otherwise require extensive procedures for curative resection, such as PD with or without hepatectomy or arterial reconstruction. In the present case, we carried out this procedure for a patient with a clinical and pathological T1 distal cholangiocarcinoma, rather than carrying out a PD with arterial reconstruction, because of the presence of the arterial anomalies. However, we acknowledge the short follow‐up duration of 21 months to adequately evaluate the oncological outcome after the minimally invasive approach for this patient.

In conclusion, pancreas‐preserving resection of the lower biliary tract, “a coring‐out technique”, appears to be feasible and less invasive than PD. This organ‐preserving approach can be an alternative procedure in selected patients.

## DISCLOSURE

Conflict of Interest: Authors declare no conflicts of interest for this article.

Author Contribution: Study conception and design: Nishida, Sugimoto, Gotohda, Konishi, Takahashi. Drafting of manuscript: Nishida, Sugimoto. Critical revision: Sugimoto, Takahashi. Final approval for publication: Nishida, Kojima, Sugimoto, Gotohda, Konishi, Takahashi.
